# The Effectiveness of the Multimodal Language Model, Google Gemini 2.5 Pro, in Solving the Specialization Exam in Gynecology and Obstetrics

**DOI:** 10.7759/cureus.95724

**Published:** 2025-10-30

**Authors:** Aleksandra Wielochowska, Aleksandra Stachowicz, Melania Olender, Ada Latkowska, Julia Glinska, Dawid Boczkowski, Dominika Radej, Anna Kowalczyk, Weronika Majchrowicz, Tomasz Dolata, Michalina Loson-Kawalec, Piotr Sawina, Wojciech Jaworski, Patrycja Dadynska

**Affiliations:** 1 Medicine, Central Teaching Hospital of the Medical University of Lodz, Lodz, POL; 2 Internal Medicine, Independent Public Provincial Integrated Hospital, Szczecin, POL; 3 Medicine, Wroclaw Medical University, Wroclaw, POL; 4 Internal Medicine, Central Teaching Hospital of Medical University of Lodz, Lodz, POL; 5 General Surgery, Specjalistyczne Centrum Medyczne, Polanica-Zdrój, POL; 6 Medicine, Wydział Lekarski, Uniwersytet Opolski, Opole, POL; 7 Internal Medicine, Wielospecjalistyczny Szpital Samodzielny Publiczny Zakład Opieki Zdrowotnej, Nowa Sól, POL; 8 Internal Medicine, Non-Public Health Care Institution (NZOZ) Hospital, Dzierżoniów, POL; 9 Medicine, Independent Public Provincial Integrated Hospital, Szczecin, POL; 10 Internal Medicine, Karol Marcinkowski University Hospital, Zielona Góra, POL

**Keywords:** artificial intelligence, chatgpt, claude, didactic tool, gemini, medical education, obst gynec, resident physicians, specialization examination

## Abstract

Background: Artificial intelligence (AI) models are developing rapidly, with growing ability to process extensive and up-to-date medical knowledge. This makes them increasingly important as didactic tools, widely used by residents preparing for the Państwowy Egzamin Specjalizacyjny (PES) or the State Specialization Examination. However, concerns remain about the accuracy and reliability of AI-generated answers. Systematic validation is therefore essential, particularly in the context of exam questions, to ensure safe and effective use in medical education. The aim of this study was to assess the educational potential of Gemini 2.5 Pro in solving the PES in gynecology and obstetrics by comparing its responses with the official key and analyzing the declared confidence level for each answer.

Materials and methods: This study was designed to empirically verify the ability of the multimodal language model Gemini 2.5 PRO to solve examination tasks at a specialist level. The research material was the PES paper in obstetrics and gynecology (spring session 2025), provided by the Center for Medical Examinations in Łódź. For the final analysis, 119 questions were qualified after one of the tasks was invalidated by the examination committee. During the exam simulation, two parameters were recorded for each question: the consistency of the model's answer with the official key and a subjective assessment of confidence expressed by the model on a 5-point scale. The collected data were used to check whether the model's effectiveness depended on the nature of the question (clinical vs. theoretical) using the chi-squared test, and to assess the correlation between confidence and the correctness of the answer using the Mann-Whitney U test.

Results: According to the observations, Gemini 2.5 PRO passed the exam with a score of 96.63%, achieving 115 points. The model's effectiveness was similar, regardless of whether the question concerned a clinical case or theoretical knowledge (p=0.313). We cannot demonstrate that the level of confidence is correlated with the effectiveness of the answer (p=0.064). When comparing the model's confidence level depending on whether the question concerned a clinical case (12 questions) or theoretical knowledge (107 questions), the difference turned out to be statistically insignificant, which means that the level of confidence was similar regardless of the question category (clinical vs. theoretical).

Conclusions: The results of the PES exam in gynecology and obstetrics clearly show that the Gemini Pro model achieved high effectiveness. A key observation was that no correlation can be demonstrated between the model's confidence level and the correctness of the answers given. Additionally, no statistically significant difference was shown in the model's confidence level between theoretical and clinical questions. The analysis proves that AI has enormous potential to support specialized education. Despite such spectacular effects, further in-depth research and constant substantive supervision by specialists are necessary to safely integrate AI with teaching programs.

## Introduction

Everyone knows ChatGPT (OpenAI, Inc., San Francisco, California, United States) [1], but it is not the only large language model on the artificial intelligence (AI) market. Alphabet Inc. (Mountain View, California, United States), which owns Google products, among others, is also active in this area. Gemini AI is an advanced artificial intelligence model developed by Google DeepMind and Google Research in response to the emergence of other AI models [2]. It is a versatile system designed for natural language processing, image analysis, coding, and many other tasks based on AI. Its main goal is to provide intelligent, precise, and contextual answers, as well as to support users in tasks requiring a creative and analytical approach. The first version of Gemini, available under the name Bard, was released in February 2023 [3]. After a short time, Google began the process of phasing out the name Bard in favor of Gemini, which was intended to emphasize a new direction in the development of AI technology, placing greater emphasis on multimodality and the ability to process different types of data [4].

Google Gemini is available in four versions: Ultra, Pro, Flash, and Nano [5]. Each of these versions has been designed for different requirements and applications, adapting to the needs of users depending on their goals and available resources. Gemini Pro, which is discussed in this study, seems to be the best model for scaling across a wide range of applications. Gemini 2.5 Pro was released on March 25, 2025. It is the latest flagship model from Google DeepMind, which combines advanced natural language processing capabilities with improved analytical abilities [6]. Unlike traditional chatbots, which work by predicting subsequent words, Gemini 2.5 actually "thinks" before answering, analyzes the context, draws conclusions, and checks its own reasoning, minimizing the risk of errors [7].

The use of advanced language models in medical education is a subject of growing interest among researchers, especially in terms of their potential to solve examination tests, such as the Państwowy Egzamin Specjalizacyjny (PES) or the State Specialization Examination [8]. The research published so far has pointed to significant achievements of AI models in this area. Błecha et al. documented that ChatGPT-4o successfully passed the Polish final exams, obtaining 71.43% on the PES in infectious diseases [9]. Furthermore, Sławińska B et al. achieved a score of 78.3% on the PES in ophthalmology in their study [10]. The AI model has not proven to be sufficiently reliable in all fields. Suwała et al. in their 2023 paper proved that the performance of ChatGPT in the internal medicine exam was insufficient and the required 60% threshold was not achieved [11].

AI is bringing revolutionary effects in medicine, changing every aspect of it, from diagnosis and treatment to the management of medical facilities. Its impact is already significant, and it will be even more transformative in the future. AI algorithms can analyze medical images, process vast amounts of laboratory and genetic data, identify disease patterns and markers, and even analyze images of tissue biopsies.

Considering the above, the aim of this study was to verify the possibility of the multimodal Gemini pro passing the PES in obstetrics and gynecology. Furthermore, the study aimed to assess whether the model's confidence level corresponded with the actual accuracy of its answers. The analysis took into account both the substantive correctness of the answers provided and the subjective assessment of the confidence with which the model gave the answers. The success criterion was achieving a threshold of 60% correct answers. The data for the analysis came from the examination questions and the answer key, published by the Center for Medical Examinations (CEM) in Łódź [8,12].

## Materials and methods

This study was designed to empirically verify the capability of the multimodal language model Gemini 2.5 PRO in solving the PES in gynecology and obstetrics. The official PES paper from the spring 2025 session, which is publicly available on the website of the CEM in Łódź [8], was used for the study. The examination initially consisted of 120 multiple-choice questions; however, one question was invalidated by the examination committee due to ambiguity, which resulted in a final set of 119 questions for the analysis.

To prepare the model for the task, it was first instructed on the rules of the examination before the simulation began. These rules consist of solving a specific pool of multiple-choice questions. This means that the respondent is tasked with selecting the correct or best answer from a given set of options (distractors). Each question consists of a so-called "stem" (a question, sentence, or phrase) and a set of possible answers.

The questions were then presented to the model sequentially by pasting them one by one into the chatbot. In the prompt for the question, we included an instruction for Gemini, which read as follows: "Based on your knowledge, answer the above question and rate your confidence level on a scale of 1 to 5, where 1 is no confidence and 5 means full confidence." We evaluated the model's results by comparing its answers, which we collected in a text file, with the official answer key available on the CEM website, and the primary success criterion was defined as achieving at least 60% (72 points) correct answers (see Appendices). Every interaction during this simulation was meticulously documented to ensure the integrity of the collected data.

For the purpose of a more granular analysis, the 119 exam questions were categorized into two distinct groups: clinical questions and theoretical questions. The clinical group was comprised of 12 questions that required the model to interpret specific patient cases to make diagnostic or therapeutic decisions. The theoretical group consisted of the remaining 107 questions, which tested general and foundational knowledge in the field. In addition to assessing the correctness of each answer, a subjective measure of the model's confidence was recorded. After providing an answer, the model was prompted to rate its confidence on a five-point scale, where 1 indicated no confidence, 2 represented low confidence, 3 signified moderate confidence, 4 corresponded to high confidence, and 5 denoted full confidence. This process allowed for a secondary analysis of the correlation between the model's self-assessed confidence and the actual accuracy of its responses.

All collected data were statistically analyzed using the Statistica software program (TIBCO Software Inc., Palo Alto, California, United States). The chi-squared test was specifically employed to compare the model's effectiveness between the clinical and theoretical question categories. To assess the relationship and differences in confidence levels between correct and incorrect answers, the Mann-Whitney U test was utilized. A p-value of less than 0.05 was established as the threshold for statistical significance in all tests. The research was conducted remotely by authors in different locations, who utilized collaborative tools such as Microsoft Teams (Microsoft Corporation, Redmond, Washington, United States), Zoom (Zoom Communications, Inc., San Jose, California, United States), Facebook Messenger (Meta Platforms, Inc., Menlo Park, California, United States), email, and Google Docs (Google LLC, Mountain View, California, United States) to ensure a comprehensive and peer-reviewed approach to the study.

## Results

**Figure 1:**
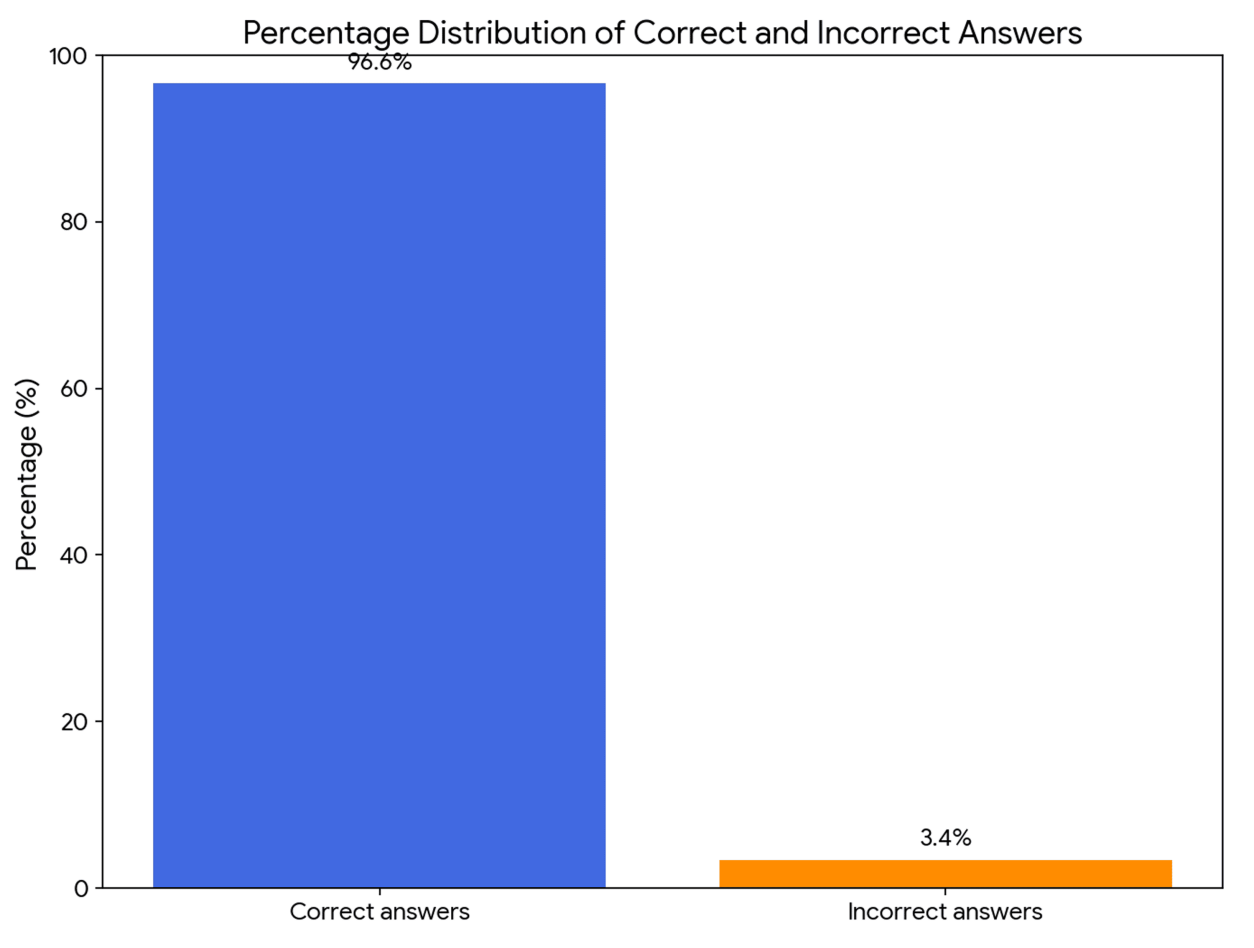
Distribution of correct and incorrect answers provided by Gemini 2.5 PRO in the obstetrics and gynecology specialization exam (N = 119).

**Figure 2:**
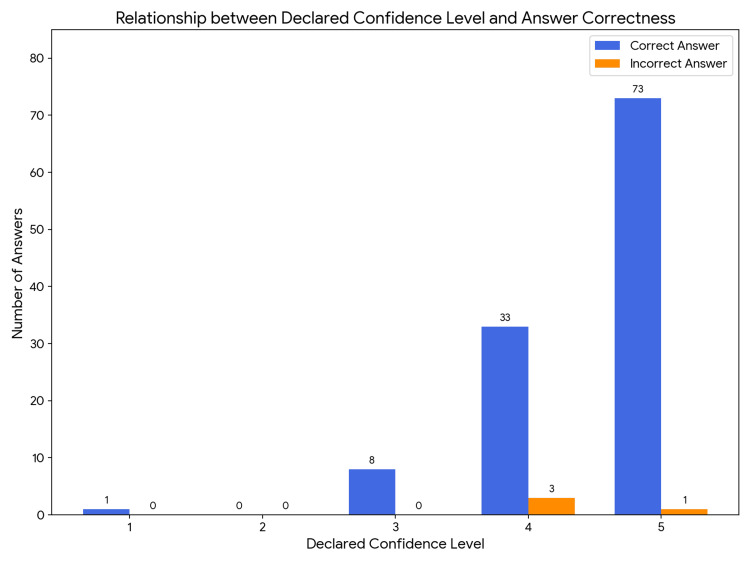
Declared confidence level in relation to the answer correctness of the Gemini 2.5 PRO model.

According to the observations, Gemini 2.5 PRO passed the exam with a score of 96.63%, achieving 115 correct and four incorrect answers (Figure 1, Table 1). Based on the data summarized in Table 2, which compares the model's effectiveness, Gemini Pro correctly answered 91.67% of questions pertaining to clinical cases and 97.19% of theoretical questions. It was concluded that the difference between these outcomes did not reach statistical significance. (p=0.313; x²=1,015). The study showed that the effectiveness of the answer is not correlated with the model's level of confidence (p=0.0639; x²=3.431) (Table 3). Based on additional data, an analysis was performed comparing the model's confidence level (on a 1-5 scale) depending on whether the question concerned a clinical case or theoretical knowledge (Figure 2). The Gemini Pro model exhibited a similar level of confidence, regardless of whether it was solving complex clinical problems or answering a theoretical question (U = 305; p = 0.119). Statistical significance was not reached. 

**Table 1:** Comparison of the answers provided by the Gemini 2.5 PRO model to the questions of the Państwowy Egzamin Specjalizacyjny (PES)* in Obstetrics and Gynecology, along with the declared confidence level. *State Specialization Examination CEM: Center for Medical Examinations

Question number	Google Gemini 2.5 PRO answer	Correct answer (CEM)	Status	Confidence (1-5)
1	A	A	Compliance	5
2	C	C	Compliance	5
3	C	C	Compliance	5
4	E	E	Compliance	5
5	B	B	Compliance	5
6	E	E	Compliance	4
7	D	D	Compliance	5
8	A	A	Compliance	5
9	B	B	Compliance	4
10	D	D	Compliance	4
11	A	A	Compliance	5
12	E	E	Compliance	5
13	C	C	Compliance	3
14	D	D	Compliance	5
15	E	E	Compliance	4
16	D	D	Compliance	5
17	A	A	Compliance	5
18	E	E	Compliance	5
19	D	D	Compliance	5
20	B	B	Compliance	5
21	E	E	Compliance	4
22	D	D	Compliance	5
23	C	C	Compliance	4
24	C	C	Compliance	4
25	D	A	Non-compliance	4
26	D	D	Compliance	5
27	B	B	Compliance	3
28	D	D	Compliance	5
29	D	D	Compliance	5
30	C	C	Compliance	4
31	B	B	Compliance	4
32	C	C	Compliance	5
33	B	B	Compliance	5
34	B	B	Compliance	5
35	A	A	Compliance	3
36	A	A	Compliance	4
37	A	A	Compliance	4
38	C	C	Compliance	4
39	x	x	x	x
40	E	E	Compliance	5
41	D	D	Compliance	5
42	C	C	Compliance	5
43	E	E	Compliance	3
44	D	D	Compliance	5
45	B	B	Compliance	5
46	C	C	Compliance	4
47	B	B	Compliance	5
48	E	E	Compliance	4
49	D	D	Compliance	5
50	E	E	Compliance	5
51	D	D	Compliance	5
52	D	D	Compliance	5
53	C	C	Compliance	5
54	E	E	Compliance	4
55	E	E	Compliance	5
56	E	E	Compliance	5
57	B	B	Compliance	4
58	A	A	Compliance	5
59	A	A	Compliance	4
60	A	A	Compliance	5
61	A	A	Compliance	5
62	A	A	Compliance	3
63	E	E	Compliance	4
64	A	A	Compliance	4
65	D	D	Compliance	5
66	E	E	Compliance	4
67	C	C	Compliance	3
68	C	C	Compliance	4
69	B	C	Non-Compliance	5
70	C	C	Compliance	5
71	C	C	Compliance	5
72	C	C	Compliance	5
73	D	D	Compliance	5
74	D	D	Compliance	4
75	C	C	Compliance	4
76	D	D	Compliance	5
77	D	D	Compliance	5
78	C	C	Compliance	5
79	C	C	Compliance	5
80	C	C	Compliance	5
81	E	E	Compliance	5
82	C	C	Compliance	5
83	A	A	Compliance	4
84	C	C	Compliance	5
85	E	C	Non-compliance	4
86	B	B	Compliance	4
87	E	E	Compliance	5
88	E	E	Compliance	4
89	B	B	Compliance	5
90	E	E	Compliance	4
91	E	E	Compliance	3
92	D	D	Compliance	4
93	C	C	Compliance	5
94	B	B	Compliance	4
95	C	C	Compliance	5
96	B	B	Compliance	5
97	E	E	Compliance	5
98	D	D	Compliance	5
99	A	A	Compliance	5
100	B	B	Compliance	5
101	C	C	Compliance	1
102	B	B	Compliance	5
103	D	D	Compliance	5
104	A	A	Compliance	4
105	D	D	Compliance	5
106	E	E	Compliance	5
107	C	C	Compliance	3
108	D	C	Non-compliance	4
109	D	D	Compliance	5
110	D	D	Compliance	5
111	E	E	Compliance	5
112	D	D	Compliance	5
113	B	B	Compliance	5
114	D	D	Compliance	5
115	C	C	Compliance	4
116	E	E	Compliance	5
117	D	D	Compliance	5
118	E	E	Compliance	4
119	A	A	Compliance	5
120	C	C	Compliance	4

**Table 2:** Evaluation of Gemini 2.5 PRO performance by question type: clinical cases versus theoretical/other Data are presented as absolute numbers (n) and percentages (%). No statistically significant differences in response accuracy were observed between clinical and theoretical/other questions (χ² = 1.015; p = 0.313). A significance level of p< 0,05 was adopted for the analysis.

Question type	Correct answer, n (%)	Incorrect answer, n (%)	p-value	χ² value
					Clinical cases	11 (91.67)	1 (8.33)	0.313	1.015
Other/ Theory	104 (97.19)	3 (2.81)							

**Table 3:** Confidence level with which Gemini 2.5 PRO provided its answers, as well as the corresponding number of correct and incorrect responses. No statistically significant differences in response accuracy were observed between clinical and theoretical/other questions (χ² = 3.431; p = 0.0639). A significance level of p< 0,05 was adopted for the analysis.

Confidence level	Correct answer	Incorrect answer	Total	p-value	χ² value
4	33	3	36	0.064	3.431
5	74	1	75		
Total	107	4	111		

Based on the data from Table 4, it can be concluded that the score of 115 points (96.63%) achieved by the Gemini 2.5 PRO model not only significantly exceeds the passing threshold of 72 points, but also surpasses the mean score (88.3), the median (91.0), and even the maximum score (109 points) achieved by physicians taking the exam for the first time. This indicates that within the conducted simulation, the AI model demonstrated higher effectiveness than the top-performing of the 111 physicians who took the exam. The table presents the model's answers in relation to the correct answers, according to the official answer key published by the CEM in Łódź. For each question, the confidence level declared by the model was included. The model assessed each question on a 5-point scale. This scale is defined as follows: 1 - no confidence, 2 - low confidence, 3 - moderate confidence, 4 - high confidence, and 5 - full confidence.

**Table 4:** Psychometric characteristics and descriptive statistics of the Państwowy Egzamin Specjalizacyjny (PES)* in Obstetrics and Gynecology (Spring Session 2025) for first-time examinees. *State Specialization Examination

Statistical Parameter	Value
Number of examinees	111
Number of examinees with complete answers	109
Mean score	88.3
Standard deviation (SD) of scores	12.28
Median score	91.0
Maximum score	109
Minimum score	48
Mean difficulty index	0.730
Mean discrimination power index	0.245
Kuder-Richardson 20 (KR20) reliability coefficient	0.898
Test passing threshold	72
Number of examinees below the threshold	10 (9.01%)

The results of 111 physicians were analyzed. The mean test score was 88.3 with a standard deviation of 12.28. The median score (91.0) was higher than the mean, indicating a slightly left-skewed distribution of scores, with the majority of examinees achieving results above the average. The high reliability of the test, estimated using the Kuder-Richardson 20 formula (KR20 = 0.898), demonstrates the strong internal consistency of the measurement tool. The passing threshold was set at 72 points, and the percentage of individuals who did not meet this threshold was 9.01%.

## Discussion

In the present study, the model achieved a score of 96.63% (115 correct answers, four incorrect), thus significantly exceeding the passing threshold. Additionally, it demonstrated an effectiveness surpassing the average results of physicians taking the same exam for the first time. Statistical analysis revealed no significant difference in performance between clinical and theoretical questions (χ²=1.015; p=0.313). The model performed comparably well regardless of the question type, on both clinical case-based tasks and questions testing purely theoretical knowledge.

This result represents significant progress compared to previous studies on the effectiveness of AI models on Polish and international medical exams. As recently as 2023, Suwała et al. demonstrated that the ChatGPT model was unable to achieve the required 60% threshold on the PES in Internal Medicine from the years 2013-2017 [11]. A 2022 study by Hanna et al. evaluated the performance of ChatGPT 4.0, ChatGPT 3.5, and Bard (an earlier version of Gemini) on the Family Medicine specialty certification exam [13]. The highest performance was demonstrated by ChatGPT 4.0, which achieved a score of 86.5%. Lower scores were recorded for ChatGPT 3.5 and Bard, both at 64.2%. All three AI models successfully passed the exam. In March 2024, a study published by Botross et al. reported that Bard obtained a score of 62.4% on the ophthalmology specialty certification exam [14]. The presented data unequivocally indicate a rapid and significant advancement in the capacity of AI to address complex medical problems.

A key point where our results differ from previous studies is the issue of self-assessment, or confidence. A statistical analysis was conducted using the Mann-Whitney U test to examine how the model self-assessed the confidence of its answers on a scale from 1 (no confidence) to 5 (full confidence). As the calculated p-value is greater than the standard significance threshold (p<0.05), there is no statistically significant difference in the confidence levels between correct and incorrect answers (U=305, p=0.199). This means that Gemini Pro did not demonstrate a capacity for "metacognition"; it was unaware of when it was providing a correct answer. This is a significant difference compared to analyses of the GPT series models. For example, in the study by Suwała et al. evaluating GPT-3.5 on the Medical Final Examination (LEK), it was observed that the model showed higher confidence when providing correct answers [15]. Similarly, in the work by Bielówka et al., where ChatGPT 3.5 scored 45.38% on the specialization exam in pathomorphology, a correlation was shown between the model's confidence and the correctness of its answers [16]. The absence of this correlation in the case of Gemini Pro is a significant finding that requires further investigation, especially in the context of potential clinical use, where a model that is "confident in its errors" could pose a threat.

Discrepancies in the results obtained in the cited works may stem from differences in the datasets on which the individual models were trained, as well as their processing capabilities. In a study conducted by Hen et al. comparing the performance of the Gemini, ChatGPT-4, and ChatGPT-3.5 models on the Taiwanese pulmonology exam from 2013-2023, the Gemini model performed slightly worse than ChatGPT-4 in terms of answer accuracy [17]. This may suggest differences in the knowledge levels between these models in specific domains.

These observations are consistent with the results from a study on the German medical exam, where Geissler et al. also noted a slight advantage of GPT-4 over Gemini Pro [18]. This may suggest differences in the knowledge levels between these models in specific, narrow domains. Nevertheless, the overall trend is clear: newer versions of AI models are characterized by higher pass rates and better results than older models, as seen in the study by Humar et al., in which they evaluated the performance of ChatGPT on the Plastic Surgery In-Service Examination and compared it with the results of residents on a national scale [19]. In total, 1129 questions were included in the final analysis, and ChatGPT answered 630 (55.8%) of them correctly. To conduct a more thorough analysis, further observations and studies of these models across various medical fields are necessary.

A critical evaluation of this study's methodology reveals several limitations that affect the interpretation of the results. First and foremost, a static evaluation method was used, with each question being posed only once. It was not investigated how multiple attempts or changes in the phrasing of the questions could have influenced the results, which is significant in the context of the probabilistic nature of the model's responses. The analysis also did not include a qualitative assessment of the errors, leaving it unknown whether they resulted from a lack of knowledge or a misinterpretation of the question. Moreover, the scope of the study was limited to a single Polish specialization exam from one session and a single language model, Gemini 2.5 Pro. This specificity narrows the generalizability of the findings to other examination systems, countries, or AI models. The potential risk that the training data may have included material overlapping with the exam content must also be considered, as this could have given the model an unfair advantage and inflated its scores. Finally, the observed lack of correlation between the model's confidence and the correctness of its answers is in itself a limitation for practical applications, casting doubt on its reliability.

## Conclusions

The Gemini 2.5 PRO model achieved an encouraging result of 96.63% in this study, exceeding the passing threshold for the specialization exam in obstetrics and gynecology (spring 2025). The obtained result should be assessed as very good. Gemini not only outperformed the average physician taking the exam for the first time, but its score also surpassed that of the top-performing human candidate. Despite its impressive effectiveness, there are discrepancies between the model's confidence and its reliability. Further work is needed to correlate the model's confidence level with the answers given and thus minimize the risk of error. This is crucial for building trust in the model and its usefulness in all fields of medicine. 

## Appendices

**Table 5:** Answer key to 120 multiple-choice questions from Państwowy Egzamin Specjalizacyjny (PES) in gynecology and obstetrics, Center for Medical Examinations, spring 2025

Questions	Answers
Question 1 Indicate the true statements regarding the process of fertilization and implantation: 1. hyaluronidase activity allows penetration of the corona radiata cells; 2. glycoprotein ZP1 is responsible for blocking polyspermy; 3. the embryo reaches the uterine cavity at the morula stage; 4. the embryonic period lasts from the second week of pregnancy to the end of the eighth week of pregnancy; 5. cortical granules are released during the so-called acrosomal reaction. The correct answer is: A. 1,3,4. B. 1,2,3,4. C. 1,3,4,5. D. 3,4,5. E. all of the above.	A
Question 2 The characteristic features of complete androgen insensitivity syndrome include: 1) primary amenorrhea during puberty; 2) partially preserved breast development; 3) hypoplastic uterus; 4) sparse pubic and axillary hair; 5) presence of testes in the inguinal canals or labia majora. The correct answer is: A. all of the above. B. 1,3,4. C. 1,2,4,5. D. 1,3,4,5. E. 1,3,5.	C
Question 3 Indicate the false statement regarding the development of ovarian follicles: A. the ovaries of female fetuses contain several million germ cells. B. in the ovaries of girls at the beginning of puberty, there are about 400,000 germ cells. C. both meiotic divisions of the oocyte are completed before ovulation. D. a primordial ovarian follicle is an oocyte covered by a layer of granulosa cells. E. a mature ovarian follicle before ovulation is called a Graafian follicle.	C
Question 4 Indicate the true statement(s) regarding congenital adrenal hyperplasia: A. in its classic form, it can lead to virilization of a female fetus. B. it is most often due to a 21-hydroxylase deficiency. C. it causes a deficiency of cortisol and mineralocorticoids. D. in the non-classic form, 17-hydroxyprogesterone (17-OHP) measurement is used for diagnosis. E. all of the above.	E
Question 5 Ovulation, the process of releasing an oocyte from the follicle: A. is preceded by a preovulatory increase in testosterone. B. is preceded by an LH surge. C. does not require the action of proteolytic enzymes. D. occurs in every cycle in women who menstruate regularly. E. occurs after the formation of the corpus luteum	B
Question 6 Factors that increase the risk of hyperprolactinemia do not include: A. therapy with serotonin reuptake inhibitors. B. regular and intense physical exercise. C. pregnancy. D. stress. E. therapy with proton pump inhibitors.	E
Question 7 Indicate the false statement concerning estrogens: A. most estradiol comes from the ovary. B. estrone is the main estrogen in the postmenopausal period. C. estrogens cause endometrial proliferation. D. they decrease the concentration of coagulation factors II, VII, IX, and X. E. they stimulate bone formation	D
Question 8 Indicate the false statement concerning prolactin: A. its concentration is not sensitive to the administration of antipsychotic and antidepressant drugs. B. it is secreted by the anterior pituitary lobe. C. in the circadian rhythm, the highest concentrations are observed at night. D. its main actions are influencing the development of the mammary gland and inducing milk production. E. it is secreted in a pulsatile manner.	A
Question 9 Factors improving the treatment outcomes of cervical cancer are: 1) a period of at least 5 years before cancer recurrence (the longer the period without recurrence, the better the outcome); 2) peripheral location of the recurrence focus; 3) size of the recurrence focus up to 2 cm; 4) diagnosis of adenocarcinoma; 5) age > 45 years. The correct answer is: A. 1,3. B. 1,2,3. C. 2,3. D. 4,5. E. all of the above.	B
Question 10 Indicate the true statements concerning the treatment of endometrial cancer: 1) according to the FIGO 2023 classification of corpus uteri cancer, for proper staging of the neoplastic process, it is required to determine the presence of metastases to the pelvic and para-aortic lymph nodes with differentiation into micro- and macrometastases; 2) macrometastases are defined as a metastatic focus with a diameter of more than 5 mm; 3) one of the criteria for the presence of micrometastases is the finding of more than 200 neoplastic cells in the examined lymph node; 4) isolated tumor cells are found when the focus is less than 0.2 mm in diameter or the number of neoplastic cells is less than 300; 5) the stage of endometrial cancer with micrometastases to the para-aortic lymph nodes is defined as stage IIIC2i. The correct answer is: A. 1,2,3. B. 1,2,4. C. 1,3. D. 1,3,5. E. 1,2,4,5.	D
Question 11 The Fagotti score is a tool that facilitates the qualification of patients with ovarian cancer for cytoreductive surgery. Which tool is necessary to assess the patient according to the Fagotti score? A. diagnostic laparoscopy. B. exploratory laparotomy. C. magnetic resonance imaging. D. computed tomography. E. ultrasonography.	A
Question 12 Indicate in which situations hormonal therapy for endometrial cancer is used: 1) fertility-sparing therapy; 2) adjuvant therapy, after brachytherapy; 3) adjuvant therapy, together with chemotherapy; 4) adjuvant therapy, after teleradiotherapy; 5) palliative therapy. The correct answer is: A. 1, 2. B. 2, 3, 4. C. 3, 4. D. 1, 2, 5. E. 1, 5.	E
Question 13 Indicate the true statements concerning prophylactic surgical treatment of patients with a hereditary predisposition to ovarian cancer: 1) in BRCA2 mutation carriers, according to NCCN guidelines, it is not recommended to postpone prophylactic surgical treatment until the age of 40-45; 2) it is indicated to collect peritoneal washings for cytological examination; 3) removal of the peritoneum from the area of ovarian or fallopian tube adhesions to the pelvic wall peritoneum is not recommended; 4) removal of the peritoneum from the ovarian fossa area, in the absence of significant surgical difficulties, should be performed via laparoscopy; 5) patients with Lynch I syndrome should undergo prophylactic bilateral adnexectomy between the ages of 40 and 45. The correct answer is: A. 1,2. B. 1,2,3. C. 2,4. D. 2,5. E. 1,2,4,5.	C
Question 14 Which specific nerves are spared in the "nerve-sparing radical hysterectomy" technique? A. obturator nerves. B. sciatic nerves. C. superior gluteal nerves. D. autonomic nerves. E. lateral femoral cutaneous nerve.	D
Question 15 Indicate the true statements concerning endometrial cancer: 1) endometrioid tumors are characterized by the immunohistochemical profile: PAX8+, CK7+, ER/PR+, vimentin+; 2) a group of patients with early endometrial cancer in whom a POLE mutation (POLEmut) is found in supplementary molecular tests belongs to the poor prognosis group; 3) immunohistochemical assessment of the expression of MLH-1, MSH-2, MSH-6, and PMS2 proteins is a tool to confirm or exclude the presence of the MMRd phenotype; 4) the group of patients with G3 endometrioid cancer is a homogeneous group in terms of prognosis and molecular profile, therefore genetic profiling is not applicable in this group; 5) in about 29% of patients with atypical hyperplasia, endometrial cancer coexists. The correct answer is: A. 1,2,3. B. 1,2,4. C. 1,3,4. D. 2,3,5. E. 1,3,5.	E
Question 16 Indicate the organs resected during a "modified posterior exenteration": A. urinary bladder and uterus. B. urinary bladder, uterus, and rectum. C. urinary bladder, vagina, uterus, and rectum. D. uterus, vagina, and rectum. E. uterus and rectum.	D
Question 17 Indicate which factor would be the most significant argument against performing a "modified posterior exenteration" in a patient with ovarian cancer: A. inability to resect neoplastic foci to NGR (no gross residual disease) or GR-1 (optimal cytoreduction). B. inability to restore gastrointestinal tract continuity. C. prior neoadjuvant chemotherapy. D. clear cell ovarian cancer. E. serous ovarian cancer.	A
Question 18 Indicate the true statements concerning granulosa cell tumor: 1) it belongs to the group of germ cell ovarian tumors; 2) it occurs bilaterally in over 15% of cases; 3) adjuvant chemotherapy is recommended in all stages of the tumor; 4) granulosa cell tumor is a neoplasm characterized by late recurrences; 5) inhibin level measurement is used to monitor treatment and detect recurrences. The correct answer is: A. 1,2. B. 1,3. C. 1,2,3,4. D. 1,2,4,5. E. 4,5.	E
Question 19 A 56-year-old patient was admitted to the gynecologic oncology ward due to a left ovarian tumor measuring 42x56 mm, accompanied by CA125 = 234 U/ml and HE4 = 157 pmol/l, with no signs of dissemination in additional imaging studies. During laparoscopy, a tumor was visualized on the surface of the left ovary. Peritoneal washings were collected, and then the affected left adnexa were removed and extracted using a laparoscopic bag. The obtained material was sent for intraoperative examination, yielding the result: endometrioid carcinoma G1, no neoplastic cells found in peritoneal washings. The uterus, right adnexa, and greater omentum were removed; biopsies were taken from the peritoneum of the abdominal cavity and lesser pelvis; and pelvic and para-aortic lymphadenectomy up to the level of the left renal vein was performed. Ultimately, apart from the described left ovarian lesion, the initial histopathological diagnosis was confirmed, and no other neoplastic foci were found. Indicate the correct postoperative management: A. Postoperative chemotherapy based on platinum compounds and paclitaxel combined with bevacizumab, which should be continued as maintenance therapy after chemotherapy is completed. B. Postoperative chemotherapy based on platinum compounds and paclitaxel. C. Given the clinical stage and histopathological type of the tumor, use of anti-angiogenic therapy in monotherapy. D. Further observation - the radical cytoreduction procedure performed is sufficient treatment, considering the clinical and histopathological stage. E. Performance of restaging, as the initially performed scope of the procedure does not allow for correct determination of the clinical stage and thus establishment of postoperative management.	D
Question 20 Indicate the false statement concerning neoadjuvant chemotherapy in ovarian cancer: A. it is chemotherapy used before surgical treatment. B. it is chemotherapy used after surgical treatment. C. neoadjuvant chemotherapy is used more often in older patients. D. neoadjuvant chemotherapy is used more often in patients with more advanced disease. E. most often, 3 or 4 courses of neoadjuvant chemotherapy are administered.	B
Question 21 Indicate the true statements concerning choriocarcinoma: 1) it can develop after both a molar pregnancy and a term pregnancy; 2) it cannot develop after an ectopic pregnancy; 3) the most common site of metastasis is the lungs; 4) brain and liver metastases occur in approx. 10% of cases; 5) patients with a score of at least 7 in the FIGO/WHO classification constitute a high-risk group and should start chemotherapy with a multi-drug regimen. The correct answer is: A. 1,2. B. 1,2,3. C. 1,3,4. D. 1,2,3,5. E. 1,3,4,5.	E
Question 22 A 65-year-old patient was admitted to the gynecologic oncology ward due to an increased abdominal circumference, lower abdominal pain, and a diagnosis of bilateral solid ovarian tumors on transvaginal ultrasound. A contrast-enhanced computed tomography scan of the pelvis, abdomen, and chest was performed. The scan revealed the presence of bilateral ovarian tumors, implants on the surface of the parietal peritoneum in all 4 quadrants of the abdominal cavity, and enlarged lymph nodes, including nodes above the renal vessels. By the decision of the multidisciplinary team, the patient was disqualified from radical surgical treatment. Indicate the most appropriate further management: A. Initiation of chemotherapy with paclitaxel and carboplatin. B. PARP inhibitor therapy. C. Anti-angiogenic therapy with bevacizumab. D. Diagnostic laparoscopy and tumor biopsy. E. Laparotomy and removal of the uterus with adnexa, leaving non-resectable lesions.	D
Question 23 Indicate the correct management in the case of a pathomorphological diagnosis of AIS (adenocarcinoma in situ) in women wishing to preserve fertility, with negative margins (after LLETZ, conization performed to exclude invasion), as part of HPV HR-based screening in women aged 25-64: A. Co-test + ECC every 6 months for 3 years, then annually for 2 years. B. HPV HR every 6 months for 3 years, then annually for 3 years. C. Co-test + ECC every 6 months for 2 years, then annually for 3 years. D. HPV HR every 6 months for 2 years, then annually for 3 years. E. Co-test + ECC every 12 months for 3 years, then annually for 5 years.	C
Question 24 Indicate the true statement concerning OHVIRA syndrome: A. it is a developmental anomaly characterized by a double uterus, one normal vagina and a second blind-ending vagina, and the absence of a kidney on the side of the normal vagina. B. 6 subtypes of the syndrome are distinguished. C. in the course of this syndrome, hematocolpos develops a few months after menarche. D. the surgical procedure involves excising one of the uteri. E. this syndrome is always diagnosed in the neonatal period.	C
Question 25 First-line therapy for endometriosis in adolescents includes the use of: 1) dienogest; 2) GnRH analogs; 3) oral hormonal contraception; 4) levonorgestrel-releasing intrauterine system; 5) progestogens. The correct answer is: A. 1,3,5. B. 1,5. C. 1,2,5. D. 3,4. E. 1,2,3,5.	D
Question 26 Uterine and vaginal agenesis occurs in the following medical conditions: 1) Mayer-Rokitansky-Küster-Hauser (MRKH) syndrome; 2) Turner syndrome; 3) Swyer syndrome; 4) OHVIRA syndrome; 5) Androgen Insensitivity Syndrome (AIS). The correct answer is: A. 1,3,5. B. 2,3,4. C. 1,4,5. D. 1,5. E. 1,2,5.	D
Question 27 The most common heart defects in fetuses of adolescent mothers include: 1) complete transposition of the great arteries; 2) tricuspid atresia/stenosis; 3) right ventricular outflow tract obstruction; 4) mitral atresia/stenosis; 5) total anomalous pulmonary venous return. The correct answer is: A. 1,3,5. B. 1,3. C. 2,3,4,5. D. 1,5. E. all of the above.	B
Question 28 In the therapy of endometriosis in adolescents, the second-line treatment is: A. dienogest. B. combined oral contraceptives. C. levonorgestrel-releasing intrauterine system. D. GnRH analogs. E. danazol.	D
Question 29 Performing a diagnostic and therapeutic laparoscopy in a woman with suspected endometriosis is indicated: A. before attempting pharmacological treatment to confirm endometriosis. B. after prior use of pharmacological treatment to improve visualization during the surgical procedure. C. in a situation of incidental finding on ultrasound of an asymptomatic cyst with the echostructure of an endometrioma in a patient undergoing an IVF-ET procedure. D. in the absence of efficacy or intolerance to pharmacological pain management and in patients unable to conceive. E. laparoscopy is not currently a method used in the diagnosis of endometriosis	D
Question 30 The decision to surgically treat extremely advanced endometriosis (frozen pelvis syndrome) is the treatment of choice in the following clinical situations, with the exception of: A. progressive hydronephrosis. B. bowel subobstruction. C. intestinal infiltration involving more than half of its circumference. D. a pain syndrome requiring chronic pharmacological treatment. E. intolerance or contraindications to pharmacological treatment.	C
Question 31 Indicate the true statements regarding the diagnosis of endometriosis: 1) in patients not currently planning pregnancy, diagnostic laparoscopy without a trial of pharmacological treatment is not recommended solely for the purpose of confirming endometriosis; 2) during diagnostic laparoscopy, it is not recommended to perform a biopsy of endometriosis foci for its confirmation, as visual confirmation is sufficient; 3) for the confirmation of endometriosis, a biopsy from unchanged peritoneum is not recommended; 4) it is recommended to perform a diagnostic and therapeutic laparoscopy in women with inability to conceive and suspected endometriosis. The correct answer is: A. 1,2,4. B. 1,3,4. C. 2,3,4. D. 3,4. E. 1,2,3,4.	B
Question 32 EFI - endometriosis fertility index is an indicator describing the probability of spontaneous pregnancy after surgery for endometriosis. This index consists of the following elements: 1) patient's age; 2) duration of infertility; 3) stage of endometriosis according to ASRM; 4) information on whether the patient was hormonally treated; 5) visual assessment of adnexal function during surgery; 6) partner's age. The correct answer is: A. all of the above. B. 1,2,3,4. C. 1,2,3,5. D. 2,3,4,5. E. 1,2,5,6.	C
Question 33 Indicate the true statements regarding pertussis (whooping cough) vaccination in a pregnant woman: 1) vaccinating a woman against pertussis to protect the newborn and infant is equally effective before pregnancy as it is during pregnancy; 2) vaccinating a woman against pertussis to protect the newborn and infant during pregnancy is more effective than before pregnancy; 3)the dTap vaccine, containing a reduced dose of diphtheria toxoid, an acellular pertussis component, and tetanus toxoid, is considered safe for pregnant women; 4) in pregnant women, the dTap vaccine... should be administered up to the 27th week of pregnancy; 5)in pregnant women, the dTap vaccine... should be administered from the 27th week to the 36th week of pregnancy. The correct answer is: A. 1,3,4. B. 2,3,5. C. 1,3,5. D. 2,3,4. E. 2,4.	B
Question 34 Recommended prophylactic measures for women with Lynch syndrome include all of the following, with the exception of: A. annual transvaginal ultrasound from age 30-35. B. annual breast ultrasound from age 30-35. C. annual CA125 measurement from age 30-35. D. colonoscopy every 1-2 years from age 20-25. E. hysterectomy with adnexectomy after age 35 or after completion of childbearing.	B
Question 35 Which of the following incorrectly indicates a gynecological examination position for women with physical disabilities? A. in women with severe spasticity in extreme situations, the lateral-knee position is recommended. B. in women with severe spasticity, with suspicion of oncological diseases, intravenous anesthesia should be used to overcome the spasticity and perform the examination on a gynecological chair in the classic position. C. in women with limited hip joint mobility, the "diamond" position is recommended. D. in disabled patients of developmental age, the "M" position is recommended. E. in women undergoing an examination on a wheelchair, the "V" position is recommended.	A
Question 36 Over the last decades, obesity has become a global epidemic and a serious public health problem. A link has been shown between maternal obesity and an increased risk of fetal defects, with the exception of: A. gastroschisis. B. neural tube defect. C. omphalocele. D. hydrocephalus. E. cleft lip and palate.	A
Question 37 Based on the PTGiP guidelines "Algorithms for screening and management of abnormal results within the Cervical Cancer Prevention Program funded by the National Health Fund - edition after the introduction of high-risk human papillomavirus diagnostics," indicate the true statements concerning pregnant women: 1) loop electrosurgical excision procedure (LEEP)/LLETZ can be performed on a pregnant woman in a hospital setting up to the 18th week of pregnancy, but the indications for this procedure are limited; 2) pregnancy is a risk factor for intraepithelial lesions and their progression; 3) histologically confirmed intraepithelial lesions of the HSIL CIN2, HSIL CIN3 type during pregnancy should be monitored every 12-24 weeks using cytological tests and colposcopy at an expert level; 4) in the case of histologically confirmed intraepithelial lesions of the HSIL CIN2, HSIL CIN3 type during pregnancy, it is permissible to postpone examinations until at least 4 weeks after delivery; 5) endocervical curettage, endometrial biopsy, as well as diagnostic excision procedures in the absence of suspicion of invasive changes, are contraindicated. The correct answer is: A. 1, 3, 5. B. 1, 4, 5. C. 2, 3, 5. D. 2, 5. E. 3, 4, 5.	A
Question 38 Indicate the true statements concerning incomplete healing of the uterine scar after a cesarean section (niche): proven individual factors for the formation of a niche after a cesarean section include the number of previous cesarean sections and the type of uterine flexion; the consequences of a niche after a cesarean section may include postmenstrual spotting, reduced fertility, cesarean scar pregnancy, and placenta accreta; the treatment of choice in an asymptomatic patient not planning pregnancy, in whom a niche is found on transvaginal ultrasound, is hysteroscopic excision of the niche edges; in a patient planning pregnancy, scar revision is indicated regardless of the presence of symptoms or a relevant obstetric history (scar dehiscence, scar pregnancy); scar revision surgeries are performed laparoscopically, vaginally, or via laparotomy. The correct answer is: A. all of the above. B. 1,2,3,5. C. 1,2,5. D. 1,2,4,5. E. 1,2.	C
Question 39 Fitz-Hugh-Curtis syndrome is: A. perihepatitis with adnexitis in women. B. a complication of gonorrhea. C. a complication of chlamydia infection. D. answers A and B are correct. E. answers A and C are correct. No clear answer for this question	x
Question 40 LUTS (Lower Urinary Tract Symptoms) in women related to the period immediately following micturition include: A. feeling of incomplete bladder emptying. B. urgency. C. post-micturition dribble. D. answers A and B are correct. E. answers A and C are correct	E
Question 41 Indicate the true statement regarding genitourinary fistulas: A. the most common type of fistula is a utero-vaginal fistula. B. Youssef's syndrome, characteristic of a vesicouterine fistula, is characterized by cyclical hematuria with apparent amenorrhea without urinary incontinence. C. surgeries for uncomplicated fistulas following gynecological procedures performed 3 months after diagnosis are less effective than surgeries performed 2-3 weeks after hysterectomy. D. the most common cause of a vesicovaginal fistula is a total hysterectomy. E. the duration of drainage after surgery for fistulas caused by radiotherapy is usually up to 7 days.	D
Question 42 Asymptomatic bacteriuria is characterized by a significant number of cultured bacteria, which is: A. ≥ 10⁵ CFU/ml in a midstream urine sample; ≥ 10³ CFU/ml in a catheter-collected urine sample. B. ≥ 10⁵ CFU/ml in a midstream urine sample; ≥ 10³ CFU/ml in a catheter-collected urine sample, any number of bacteria cultured from urine obtained by suprapubic aspiration. C. ≥ 10⁵ CFU/ml in a midstream urine sample; ≥ 10² CFU/ml in a catheter-collected urine sample, any number of bacteria cultured from urine obtained by suprapubic aspiration. D. ≥ 10⁵ CFU/ml in a midstream urine sample; ≥ 10⁴ CFU/ml in a catheter-collected urine sample, any number of bacteria cultured from urine obtained by suprapubic aspiration. E. ≥ 10⁵ CFU/ml in a midstream urine sample; ≥ 10⁴ CFU/ml in a catheter-collected urine sample, ≥ 10² CFU/ml in urine from a suprapubic aspiration.	C
Question 43 Indicate the false statements concerning vaginal hysterectomy: 1) when choosing the surgical technique, the number of fibroids is of the greatest importance; 2) a uterine size not exceeding 16 weeks is an optimal indication for vaginal hysterectomy; 3) if the angle between the lateral borders of the uterus is less than 140 degrees, it facilitates the removal of the uterus through the vagina; 4) in case of an absolute necessity to remove the adnexa during hysterectomy, a laparoscopic approach or assistance should be considered; 5) vaginal hysterectomy of a uterus with large subserosal fibroids located on the anterior wall is usually technically more difficult than removing a uterus with fibroids on the posterior wall. The correct answer is: A. only 1. B. 3, 4. C. 1, 2, 3. D. 2, 4, 5. E. 1, 3, 4, 5.	E
Question 44 A 45-year-old patient presented to the gynecological clinic due to heavy menstrual periods, causing anemia (HGB 10.2 g/dl). The patient's history includes: 2 natural childbirths and an appendectomy complicated by diffuse peritonitis. Gynecological examination revealed a myomatous uterus corresponding in size to a 17-week pregnancy. After uterine curettage, histopathological examination revealed complex endometrial hyperplasia without atypia. Indicate the correct further management: A. 3-month hormonal treatment with progestogens, followed by a repeat control uterine curettage. In case of no regression of the hyperplasia, qualification of the patient for hysterectomy. B. 12-month hormonal treatment with progestogens, followed by a repeat control uterine curettage. In case of no regression of the hyperplasia, qualification of the patient for hysterectomy. C. vaginal hysterectomy without a trial of hormonal treatment. D. due to the very high risk of progression to endometrial cancer and anemia, the optimal and safest treatment for the patient would be the quickest possible abdominal hysterectomy. E. due to the low risk of malignancy, the patient does not require treatment, only control curettage and transvaginal ultrasound every 6 months.	D
Question 45 The gold standard in the diagnosis of chronic endometritis is: A. a positive result of microbiological cultures from an endometrial biopsy sample. B. stromal edema with the presence of CD138+ plasma cells in the histopathological examination of an endometrial biopsy sample. C. hysteroscopic visualization of the uterine cavity. D. the presence of immune complexes binding the complement component C1q in an endometrial biopsy sample. E. answers B and D are correct.	B
Question 46 Indicate the true statements regarding the pharmacological treatment of ectopic pregnancy: 1) the multi-dose regimen consists of administering methotrexate intramuscularly at a dose of 1 mg/kg body weight on days 1, 3, 5, 7 of treatment and folinic acid (0.1 mg/kg body weight) on days 2, 4, 6, 8 of treatment; 2) the single-dose regimen consists of an intramuscular injection of methotrexate at a dose of 50 mg/m² and does not require folinic acid supplementation; 3) the single-dose regimen has lower efficacy than the multi-dose regimen; 4) the single-dose regimen is associated with fewer adverse effects and is better tolerated by patients; 5) if on the 7th day of single-dose therapy, the β-hCG level drops by less than 15%, another dose of the drug should be administered. The correct answer is: A. 1,2. B. 1,2,3,5. C. 1,3,4,5. D. 1,3,4. E. all of the above.	C
Question 47 Indicate the true statement concerning emergency contraception: A. emergency contraception is a method that involves using levonorgestrel or ulipristal acetate before every act of intercourse. B. the contraceptive mechanism of action of levonorgestrel and ulipristal acetate is to inhibit the LH surge, inhibit/delay ovulation, and block the maturation of the dominant follicle in the ovary. C. levonorgestrel and ulipristal acetate increase the risk of ectopic pregnancy. D. levonorgestrel is a preparation that can be used up to 120 hours after intercourse, and ulipristal acetate up to 72 hours. E. levonorgestrel more often causes adverse effects compared to the preparation containing ulipristal acetate.	B
Question 48 Indicate the true statement concerning nonspecific vaginal infections: A. the most commonly diagnosed are MCU infections or non-Candida type fungi. B. polyhexamethylene biguanide in the treatment of NSV/BV is characterized by inhibiting the metabolism of bacterial cells, having higher efficacy in a low pH environment. C. povidone-iodine with octenidine and silver in the form of vaginal globules is the most effective, though forgotten, treatment regimen for BV. D. chlorhexidine, due to its high bactericidal activity in treating infections of certain pathogens, such as methicillin-resistant S. aureus, as well as P. aeruginosa and A. baumannii, is the first-line treatment for NSV. E. boric acid in the form of vaginal globules is particularly effective in treating vaginitis caused by fungi of the species C. glabrata and C. krusei.	E
Question 49 The mechanism of action of a copper intrauterine device consists of: A. the uterus's reaction to a foreign body - edema, hyperemia, accumulation of leukocytes. B. the impairing effect of copper ions on sperm motility. C. preventing the implantation of the embryo. D. answers A and B are correct. E. answers A and C are correct.	D
Question 50 A 32-year-old patient trying to conceive was diagnosed with a 5 cm diameter fibroid in the anterior uterine wall. It was classified as type 3 according to the FIGO 2011 classification. The patient has never been pregnant, has never had surgery, and is not being treated for any comorbidities. Indicate the correct further management: A. use only pharmacological treatment, as surgical removal of fibroids, during which the uterine cavity may be opened, is contraindicated in patients who have not completed childbearing. B. perform hysteroscopy with fibroid resection; attempts to conceive can begin no earlier than 3 months from the date of the procedure. C. perform hysteroscopy with fibroid resection; attempts to conceive can begin no earlier than 6 months from the date of the procedure. D. perform laparoscopy with myomectomy; if the uterine cavity was opened, attempts to conceive can begin no earlier than 3 months from the date of the procedure. E. perform laparoscopy with myomectomy; if the uterine cavity was opened, attempts to conceive can begin no earlier than 6 months from the date of the procedure.	E
Question 51 Pharmacological treatment of uterine fibroids: A. is considered a standalone treatment method because it allows avoiding surgery. B. is recommended to prevent the growth of fibroids during pregnancy. C. is absolutely contraindicated. D. aims to alleviate symptoms associated with fibroids but does not cure them completely. It is used as preparation for surgery, not as a standalone treatment method. E. must always precede surgical treatment because it reduces the volume of fibroids by at least 50% and facilitates the course of the operation.	D
Question 52 Aerobic vaginitis is distinguished from anaerobic vaginosis by the presence of: A. a "fishy" odor. B. "clue cells". C. numerous lactobacilli. D. polymorphonuclear leukocytes. E. none of the above.	D
Question 53 Absolute contraindications to uterine artery embolization in the treatment of uterine fibroids do not include: A. pregnancy. B. severe coagulation disorders. C. BMI above 25 kg/m². D. vaginitis. E. a solid-cystic adnexal tumor.	C
Question 54 Which of the following drugs used in the pharmacological therapy of overactive bladder syndrome prolongs the QT interval, which limits its use in patients with electrolyte imbalances? A. mirabegron. B. tolterodine. C. trospium. D. fesoterodine. E. solifenacin.	E
Question 55 The components of Meigs' syndrome include: A. a solid ovarian tumor. B. fluid in the pericardium. C. fluid in the pleura and abdominal cavity. D. answers A, B, and C are correct. E. answers A and C are correct.	E
Question 56 Indicate the type of vaccines containing inactivated pathogens or their fragments that cannot be administered to pregnant women: A. vaccines containing mRNA fragments. B. vaccines containing toxoids. C. polysaccharide vaccines. D. recombinant vaccines. E. all of the above types of vaccines are permitted for use in pregnancy.	E
Question 57 Which answer indicates a clinical condition/situation in which it is necessary to perform a hysterectomy via laparotomy (Abdominal Hysterectomy - AH) instead of vaginally (Vaginal Hysterectomy - VH) or laparoscopically (Total Laparoscopic Hysterectomy - TLH)? A. lack of access to a hybrid operating room that would allow for the assistance of an interventional radiologist in case of complications. B. size of a myomatous uterus corresponding to the size of a pregnant uterus at >24 weeks of gestation. C. cervical carcinoma in situ (CIS). D. lack of experience in vaginal and laparoscopic surgeries. E. serous endometrial cancer, clinical stage 3A2 according to FIGO 2023.	B
Question 58 A 53-year-old patient, last menstrual period 2 years ago, presents to the gynecologist due to contact bleeding for 6 months and foul-smelling discharge for 3 months. P3 (3 spontaneous vaginal deliveries), A0. Medical history is negative; she has had periodic gynecological check-ups every few years. Generally healthy, with no comorbidities. She has smoked 10 cigarettes a day for 35 years. Last gynecological visit was one year ago. Pap smear at that time was normal. BMI 23 kg/m². On bimanual examination: vulva macroscopically normal, vaginal mucosa normal on speculum examination, in the posterior vaginal fornix there is brownish, sero-purulent discharge with blood. On the ectocervix, a nodular, pale-red erythroplakia is visible, which bleeds on contact. Uterus is of normal size, anteverted, anteflexed, mobile, non-tender, of normal consistency. In the adnexal regions, normal ovaries are palpable, with no pathological masses. In this case, one should: A. refer the patient to a colposcopy clinic urgently. B. take a Pap smear and schedule a follow-up visit to discuss the results. C. take a Pap smear and a vaginal swab for culture, and schedule a follow-up visit to discuss the results and potential further treatment. D. prescribe broad-spectrum vaginal suppositories and schedule a Pap smear for one week after completing the treatment. E. collect a sample for an HR-HPV test and cytology, and schedule a follow-up visit to discuss the results.	A
Question 59 Abdominal hysterectomy is the first-choice technique for a patient qualified for total uterine removal: A. in the case of a uterus with a size corresponding to a uterus greater than 21 weeks of gestation. B. in atypical endometrial hyperplasia. C. with suspected adenomyosis. D. in recurrent endometrial polyps. E. in a woman with abnormal uterine bleeding and a uterus size of 14-16 weeks gestation.	A
Question 60 One of the clinical management options for an ectopic pregnancy diagnosis is pharmacological therapy with methotrexate. Indicate the contraindications for its use: 1) bone marrow hypoplasia; 2) immunodeficiency; 3) peptic ulcer disease; 4) renal failure; 5) breastfeeding; 6) liver cirrhosis. The correct answer is: A. all of the above. B. 1, 3, 4. C. 2, 5, 6. D. 2, 3, 4, 6. E. 1, 3, 5.	A
Question 61 The Johnson maneuver is: A. a maneuver used in case of uterine inversion, which consists of attempting to reposition the uterine fundus by manually returning it to its place. B. a first-degree surgical treatment for postpartum hemorrhage, which involves ligating the descending branches of the uterine arteries via a vaginal approach. C. a second-degree surgical treatment for postpartum hemorrhage, which involves placing a "brace" suture that folds the muscle and reduces the volume of the uterine cavity. D. a bimanual maneuver used for uterine atony, which involves maintaining uterine contraction by applying simultaneous pressure through the abdominal wall and the vaginal fornix. E. a maneuver used for uterine atony, which involves manual compression of the abdominal aorta through the abdominal wall.	A
Question 62 Indicate the true statements regarding vaginal hysterectomy: 1) in patients with a history of pelvic inflammatory disease, when difficulties in opening the anterior and/or posterior cul-de-sacs can be anticipated, tissues should be dissected bluntly as close to the uterus as possible, thus minimizing the risk of injury to the bladder or large intestine; 2) routine prophylactic procedures for pelvic organ prolapse are not recommended during vaginal hysterectomy; 3) in the case of significant anterior vaginal wall prolapse (POP-Q>2), placing McCall sutures is recommended; 4) in the case of cervical dysplasia, the preferred route for hysterectomy is laparoscopic hysterectomy; 5) the hydrodissection solution used for injecting the vaginal walls may contain 0.1% adrenaline added to saline, 1% or 2% lignocaine, or 0.5% bupivacaine. The correct answer is: A. 2, 5. B. 1, 2, 5. C. 1, 2, 3. D. 2, 4, 5. E. 2, 3, 5.	A
Question 63 Indicate the true statements regarding Wilson's disease in pregnancy: 1) a typical feature of this disease is a low plasma ceruloplasmin concentration; 2) the presence of a Kayser-Fleischer ring is one of the diagnostic methods; 3) during pregnancy, the doses of medications used for Wilson's disease should be reduced by 25-50%; 4) it can lead to liver cirrhosis; 5) this disease is the result of an autosomal recessive mutation. The correct answer is: A. 1, 2, 3. B. 1, 2, 4, 5. C. 1, 2, 5. D. only 3. E. all of the above.	E
Question 64 Maternal obesity increases the risk of: 1) post-term pregnancy; 2) pre-eclampsia (PE); 3) fetal growth restriction (hypotrophy); 4) twin pregnancy; 5) postpartum depression. The correct answer is: A. all of the above. B. 1, 3, 5. C. 2, 3. D. only 3. E. 2, 4.	A
Question 65 A 32-year-old primigravida patient at 28/29 weeks of gestation presented to the Obstetrics and Gynecology Emergency Room due to decreased fetal movements for several days. On admission, blood pressure (BP) was ~160/110 mmHg, with visual disturbances and headache. Laboratory results: AST ~ 60 IU/L, ALT ~ 120 IU/L, Hgb ~ 12.5 g/dl, PLT ~ 98,000/ml, protein present in urinalysis. A pregnancy ultrasound was performed, which found: EFW ~ 990 g (2nd percentile), AEDV (Absent End-Diastolic Velocity) in the umbilical artery. Determine the stage of FGR: A. I. B. II. C. III. D. IV. E. the stage of FGR cannot be determined based on the above data.	D
Question 66 A 38-year-old woman presents for her first prenatal visit. This is her first pregnancy at 5+4 weeks according to her LMP. The patient has been treated with metformin for type 2 diabetes for 2 years and is also taking a supplement containing 400 µg of 5-MTHF and 400 µg of folic acid. Her BMI calculated during this visit is 38 kg/m², and her HbA1C level (tested 7 days before the visit) is 5.9%. The recommendations for the patient should include: A. discontinuing metformin, performing a 75g oral glucose tolerance test, and starting 150 mg of acetylsalicylic acid. B. discontinuing metformin, performing a 75g oral glucose tolerance test, and starting a prophylactic dose of low-molecular-weight heparin. C. starting insulin therapy, monitoring blood glucose levels, and starting a prophylactic dose of low-molecular-weight heparin. D. continuing metformin, performing a 75g oral glucose tolerance test, and starting 150 mg of acetylsalicylic acid. E. continuing metformin, monitoring blood glucose levels, and starting a prophylactic dose of low-molecular-weight heparin.	E
Question 67 Indicate the true statements regarding HELLP syndrome: 1) about 30% of patients with eclampsia will develop HELLP syndrome; 2) maternal complications of HELLP syndrome include, among others, pericarditis; 3) the preferred marker for the severity of hemolysis is the measurement of the level of hemoglobin-dependent transport protein (haptoglobin); 4) class 3 HELLP according to the Mississippi classification is characterized by the following laboratory values: PLT < 50,000/mm³; AST at least 70 IU/l; LDH at least 600 IU/l; 5) the risk of severe complications of HELLP syndrome increases, among other factors, if the plasma uric acid concentration is >7.8 mg/dl. The correct answer is: A. 1, 2, 3, 4. B. 3, 4, 5. C. 1, 2, 3, 5. D. 1, 3, 4, 5. E. all of the above.	C
Question 68 Which of the answers indicates a diagnosis from an ophthalmological consultation that is associated with a high risk of ocular complications in the case of a vaginal delivery? A. myopia without macular changes. B. status post laser refractive surgery if the post-procedure corneal thickness is less than 600 μm. C. status post laser refractive surgery if the post-procedure corneal thickness is less than 350 μm. D. retinal detachment operated on 1-3 years before delivery with complete retinal reattachment. E. any retinal detachment operated on in the period less than 5 years before delivery.	C
Question 69 TAPS (Twin Anemia-Polycythemia Sequence) is one of the complications in monochorionic twin pregnancies. Indicate the false statements: 1) it is a very common complication of twin pregnancy; 2) the anemia-polycythemia sequence is distinguished from TTTS (Twin-to-Twin Transfusion Syndrome) by the absence of the polyhydramnios-oligohydramnios sequence; 3) prenatal diagnosis of TAPS is based on measurements of the peak systolic velocity in the middle cerebral artery in only one of the fetuses; 4) similar to TTTS, the causal treatment is laser photocoagulation of the communicating vessels; 5) the symptomatic treatment method is intrauterine transfusion to the anemic recipient. The correct answer is: A. 1, 2. B. 1, 3, 5. C. 2, 4. D. 2, 3. E. 1, 2, 5.	B
Question 70 An obese pregnant woman was admitted to the hospital, reporting severe abdominal pain that had intensified over the last several hours. The pregnant woman works in a grocery store. Her last visit to her gynecologist was three weeks ago. During the ultrasound examination at that time, the doctor noted the presence of transudative fluid in the fetal peritoneal cavity and subcutaneous tissue, as well as a slightly enlarged placenta. She is in the 27th week of her first pregnancy and has recently noticed a significant increase in her abdominal circumference. Until now, the pregnancy had been progressing normally. Indicate the probable cause of these symptoms: A. excessive activity and physical labor of the patient. B. undiagnosed gestational diabetes. C. non-immune hydrops fetalis. D. undiagnosed toxoplasmosis infection. E. urinary tract infection.	C
Question 71 The most common indications for myomectomy include all of the following, EXCEPT: A. pain. B. excessive uterine bleeding. C. cancer or suspicion of cancer. D. problems with conceiving. E. pressure on adjacent organs, such as the bladder or rectum.	C
Question 72 Indicate the true statement regarding intra-amniotic infection (chorioamnionitis): A. the newborn's condition at birth always correlates with the severity of the maternal infection. B. intra-amniotic infection during labor does not increase the risk of neurological complications in the newborn. C. on an intrapartum CTG trace, tachycardia with reduced variability and lack of cyclicity may indicate an intra-amniotic infection. D. a sinusoidal pattern is not characteristic of severe fetal hypoxia in the course of chorioamnionitis. This type of pattern only occurs in cases of severe rhesus isoimmunization. E. in the case of maternal fever during labor, the CTG trace always shows a slight bradycardia.	C
Question 73 Indicate the test performed on a fetal sample that is not relevant in the diagnosis of non-immune hydrops fetalis: A. PCR for CMV. B. PCR for parvovirus B19. C. total protein and albumin. D. indirect Coombs test. E. virological tests for TORCH.	D
Question 74 In patients with obesity: 1) it is permissible to continue medications used for obesity therapy until the end of the first trimester of pregnancy; 2) in the situation of prior bariatric surgery, it is recommended to delay conception for 6-12 months; 3) preconceptionally, folic acid supplementation at a dose of 800 micrograms is recommended, including 400 micrograms in the form of active folates; 4) in preconception supplementation, the use of myo-inositol preparations should be considered to reduce insulin resistance and prevent neural tube defects in the fetus; 5) a 75g oral glucose tolerance test should be recommended during the first prenatal visit. The correct answer is: A. 1, 2, 5. B. 3, 4, 5. C. 1, 3, 4. D. 2, 4, 5. E. all of the above.	D
Question 75 A 32-year-old patient, status post term cesarean section (3 years prior) for failure to progress, presents to the Emergency Room of a tertiary referral center at 30 weeks of gestation due to lower abdominal pain. The patient has not had regular antenatal care (last visit at 12 weeks of gestation). The CTG trace is reactive, with no uterine contractions. The cervix is slightly shortened, external os is closed. On ultrasound assessment, the admitting physician, in addition to confirming normal fetal growth and well-being, identifies a complete placenta previa without sonographically detectable features of abnormal implantation. Cervical length on transvaginal ultrasound is 33 mm. Indicate the appropriate management: A. immediate administration of tocolysis and steroids to stimulate fetal lung maturity, followed by hospitalization until a planned cesarean section at 35 weeks of gestation if no clinical symptoms arise. B. continued hospitalization until a planned cesarean section at 36 weeks of gestation if no clinical symptoms arise. C. continued hospitalization until a planned cesarean section at 37 weeks of gestation if no clinical symptoms arise. D. outpatient management is possible until 34 weeks of gestation, then hospitalization and a planned cesarean section at 37/38 weeks of gestation if no clinical symptoms arise. E. outpatient management is possible until 37 weeks of gestation, then hospitalization and a planned cesarean section at 37/38 weeks of gestation if no clinical symptoms arise.	C
Question 76 Indicate the true statements regarding the combined test: 1) it should be offered to every pregnant woman regardless of age; 2) in the absence of abnormalities on ultrasound assessment, the double test (measurement of free beta-hCG and PAPP-A) can be omitted; 3) its valuable supplement is the triple test in the early second trimester; 4) if the combined test shows a risk of trisomy 21 of 1:350, cell-free fetal DNA testing from maternal blood is recommended. The correct answer is: A. 1, 2, 4. B. 1, 2, 3. C. 2, 3, 4. D. 1, 4. E. all of the above.	D
Question 77 A primigravida patient presents to the obstetrician-gynecologist at 7 weeks of gestation, concerned about an abnormal hemoglobin level on her complete blood count: Hb 9 g/dl. Her history includes systemic lupus erythematosus with the presence of anti-SS-A (Ro 52) and SS-B antibodies. The patient is currently in remission and taking sulfasalazine. Further iron studies yielded the following results: MCV (82 fl), serum ferritin (200 mcg/l), total iron-binding capacity (TIBC) - decreased, transferrin saturation (TSAT) - decreased, serum iron level - mildly decreased. Indicate the most likely diagnosis: A. microcytic anemia. B. megaloblastic anemia. C. folic acid deficiency anemia. D. anemia of chronic disease. E. anemia secondary to sulfasalazine therapy.	D
Question 78 Indicate the correct principles for measuring blood pressure in pregnant women in a clinical setting: 1) the patient should be in a sitting position, with her back supported, arm supported, with the antecubital fossa at the level of the 4th intercostal space; 2) the cuff should be at heart level regardless of the patient's body position; 3) the first measurement on both upper limbs, subsequent measurements on the arm where the higher pressure value was measured; 4) the use of automatic blood pressure monitors is not recommended. The correct answer is: A. 1, 2, 4. B. 1, 2, 3. C. 1, 3. D. 1, 4. E. all of the above.	C
Question 79 The most serious complications of therapeutic cordocentesis (intrauterine transfusion) include all of the following, EXCEPT: A. circulatory volume overload as a result of transfusing too large a volume of blood. B. occlusion of the umbilical vessel following extravascular administration of blood - into Wharton's jelly. C. reflex bradycardia in the mother. D. bleeding into the amniotic fluid. E. intrauterine fetal death.	C
Question 80 Indicate the true statement regarding the congenital pulmonary airway malformation volume ratio (CVR): A. it has practical application in congenital diaphragmatic hernia. B. it is calculated according to the following formula: CVR = height × anteroposterior dimension × transverse dimension × 0.25 (constant) / fetal head circumference. C. values above 1.6 are associated with a higher risk of hydrops fetalis. D. with high CVR values, the percentage of fetuses with hydrops reaches 99%. E. 3D imaging is necessary for CVR calculation.	C
Question 81 Indicate how the baseline fetal heart rate changes during a physiological pregnancy: 1) the baseline fetal heart rate remains unchanged throughout the pregnancy; 2) the baseline fetal heart rate gradually slows down towards the end of the pregnancy; 3) before the 5th week of gestation, the baseline fetal heart rate is approximately 150 beats per minute; 4) between the 9th and 10th week, the baseline fetal heart rate is approximately 170 beats per minute; 5) around the time of delivery, the baseline fetal heart rate is approximately 130 beats per minute. The correct answer is: A. only 1. B. 2, 3. C. 1, 3. D. 1, 5. E. 2, 4, 5.	E
Question 82 Indicate the true statements regarding the variability of the fetal heart rate during cardiotocograph analysis: 1) variability does not change with the progression of pregnancy and the maturation of the fetal central nervous system (CNS); 2) in a situation of insufficient oxygenation of the fetal CNS, a reduction in variability may occur; 3) undulating variability (a sinusoidal pattern) indicates fetal hypoxia; 4) absent variability can be caused by metabolic acidosis in the pregnant woman; 5) absent variability can indicate that the fetus is in a deep sleep state. The correct answer is: A. 1, 2. B. 1, 2, 3. C. 2, 4, 5. D. 1, 5. E. 3, 4.	C
Question 83 Indicate the true statements regarding subacute hypoxia in the second stage of labor: 1) it is most often the result of uterine hyperstimulation; 2) it develops over several minutes; 3) on the CTG trace, successive decelerations are visible, the total duration of which is shorter than the periods between contractions and decelerations; 4) during subacute hypoxia, the umbilical artery blood pH decreases by 0.01 every 2-3 minutes; 5) during subacute hypoxia, the umbilical artery blood pH decreases several times faster than in the case of acute hypoxia. The correct answer is: A. 1, 4. B. 1, 2, 3. C. 2, 4, 5. D. 1, 5. E. 3, 4.	A
Question 84 A doctor caring for a patient with diabetes should inform her that diabetes: A. is an absolute contraindication to the use of hormonal contraception. B. is an absolute contraindication to the use of an intrauterine system with a progestogen component. C. is not a contraindication to oral hormonal contraception, and patients should be assessed for standard contraindications to hormonal contraception and should be able to choose their preferred, effective contraceptive method, being aware of the risks associated with an unplanned pregnancy. D. with the presence of retinopathy is an indication only for natural family planning. E. type 2 is an absolute indication for the use of contraceptive pills with a content higher than 35 µg of ethinylestradiol.	C
Question 85 High-risk factors for developing pre-eclampsia in an obese pregnant woman include: 1) first pregnancy; 2) age >40 years; 3) hypertension in a previous pregnancy; 4) type 2 diabetes; 5) interpregnancy interval <10 years. The correct answer is: A. 1, 3. B. 2, 3, 5. C. 3, 4. D. 1, 4, 5. E. 2, 3, 4	E
Question 86 Indicate the etiological factors of fetal growth restriction: 1) autoimmune diseases; 2) velamentous cord insertion; 3) trisomy 21; 4) Perlman syndrome; 5) type 1 diabetes with vascular complications. The correct answer is: A. 1, 2, 3. B. 1, 2, 3, 5. C. 1, 3. D. 3, 4, 5. E. none of the above.	B
Question 87 Indicate the clinical conditions that allow for the diagnosis of severe pre-eclampsia (PE): 1) renal failure; 2) fetal growth restriction; 3) blood pressure values greater than 160/110 mmHg; 4) worsening proteinuria; 5) pulmonary edema. The correct answer is: A. 1, 2. B. only 4. C. 1, 3, 4. D. 1, 2, 3, 4. E. all of the above	E
Question 88 Indicate the clinical signs of intrauterine fetal death in a twin pregnancy during the second and third trimesters: 1) sudden resolution of pre-eclampsia symptoms; 2) sudden onset of pre-eclampsia symptoms; 3) resolution of TTTS symptoms; 4) rupture of fetal membranes; 5) onset of uterine contractions. The correct answer is: A. 1, 3, 4. B. 1, 2, 3, 4. C. 1, 3, 4, 5. D. 3, 4, 5. E. all of the above.	E
Question 89 The most common inherited thrombophilias include Factor V Leiden mutation and prothrombin G20210A gene mutation. Which of the following pregnancy complications is not associated with the presence of inherited thrombophilias? A. recurrent miscarriages. B. hyperemesis gravidarum. C. pre-eclampsia (PE). D. preterm birth. E. placental abruption.	B
Question 90 Indicate the signs that do not belong to the group allowing for the diagnosis of AFLP (Acute Fatty Liver of Pregnancy) according to the Swansea criteria: 1) encephalopathy; 2) ascites; 3) thrombocytopenia; 4) elevated transaminases; 5) hyperglycemia; 6) microvesicular steatosis on liver biopsy; 7) leukocytosis. The correct answer is: A. 1, 2, 6, 7. B. 1, 2, 5, 6. C. 1, 2, 3. D. 3, 5. E. none of the above.	E
Question 91 Indicate the factors predisposing to the formation of conjoined twins: 1) excessive alcohol consumption; 2) underweight; 3) reduced activity of the Lefty gene; 4) living in Africa or India; 5) a twin pregnancy occurring after long-term use of oral contraceptives. The correct answer is: A. 1, 2, 3. B. 1, 2, 4, 5. C. 1, 3. D. only 3. E. all of the above.	E
Question 92 Indicate the true statements regarding pregnancy-induced hypertension (PIH): 1) the first-line antihypertensive drugs in pregnant women are methyldopa, labetalol, and nifedipine; 2) the diagnosis of hypertension in pregnancy should be confirmed by out-of-office measurements or during two separate visits to the attending physician; 3) the target blood pressure values to aim for during antihypertensive therapy are 110-139/81-95 mmHg; 4) the use of loop diuretics in antihypertensive treatment is not recommended; 5) if a blood pressure value higher than 160/110 mmHg is found, the pregnant patient should be referred to the hospital. The correct answer is: A. 1, 2. B. only 4. C. 1, 3, 4. D. 1, 2, 4, 5. E. 1, 3.	D
Question 93 Indicate the true statements regarding twin pregnancy: 1) in case of a discrepancy in estimated due dates calculated from the CRLs of both fetuses exceeding 7 days, the due date calculated from the smaller fetus's CRL should be used; 2) the presence of the lambda sign is characteristic of a monochorionic twin pregnancy; 3) if chorionicity cannot be definitively determined, it should be assumed that the pregnancy is monochorionic; 4) based on the presence of the lambda or tau sign, it is possible to determine the chorionicity of the pregnancy with 95% sensitivity, and this is highest before the 14th week of gestation; 5) in a monochorionic pregnancy, ultrasound scans are recommended every 2 weeks from the 16th week, and in a dichorionic pregnancy, every 4 weeks from the 16th week. The correct answer is: A. 1, 2, 3. B. 1, 3, 4. C. 3, 4. D. 4, 5. E. 2, 3, 5.	C
Question 94 A key point in the care of a woman with epilepsy of childbearing age is pregnancy planning. Effective contraception is possible for women who are not planning a pregnancy at a given time in their lives. Indicate the false statement: A. combined contraception, regardless of the route of administration, can increase the risk of epileptic seizures up to 7-fold. B. the use of lamotrigine reduces the concentration of ethinylestradiol by about 30%, but does not affect the concentration of the progestogen. C. medroxyprogesterone acetate should be administered intramuscularly every 8-10 weeks. D. benzodiazepines do not affect the effectiveness of contraception. E. progestogen-only contraception is the recommended form of hormonal contraception for patients with epilepsy.	B
Question 95 A patient, G4 P3, at 34 weeks of gestation, was brought by the Emergency Medical Services to the obstetric emergency room. The history gathered by the EMS team: one hour ago, severe abdominal pain, syncope, heavy vaginal bleeding, increased uterine tone. Blood loss was estimated by the doctor at 1800 ml. In a patient with such severe hemorrhage, the following clinical signs can be observed, EXCEPT: A. thready pulse. B. pale skin. C. drowsiness. D. tachypnea. E. systolic blood pressure of 70-80 mmHg.	C
Question 96 Indicate the first-choice antihypertensive drugs used in the emergency treatment of hypertension in pregnant women: 1) nifedipine administered p.o.; 2) hydralazine administered i.v.; 3) urapidil administered i.v.; 4) labetalol administered i.v.; 5) nitroglycerin administered i.v.; 6) labetalol administered p.o. The correct answer is: A. 3, 4. B. 1, 2, 4. C. 1, 6. D. 3, 5. E. 2, 3.	B
Question 97 In the case of PPROM (Preterm Prelabour Rupture of Membranes): A. one should always aim to administer 2 doses of betamethasone 12 mg 24 hours apart or 4 doses of dexamethasone 12 hours apart. B. one should always aim to administer 2 doses of dexamethasone 12 mg 24 hours apart or 4 doses of betamethasone 12 hours apart. C. one should always aim to administer 2 doses of dexamethasone 12 mg 24 hours apart or 4 doses of betamethasone 12 hours apart only up to 34+0 weeks of gestation. D. a full course of corticosteroids is not indicated if intrauterine infection is diagnosed. E. one should always aim to administer 2 doses of betamethasone 12 mg 24 hours apart or 4 doses of dexamethasone 12 hours apart in women up to 36+6 weeks of gestation in the absence of signs of intrauterine infection, and therefore, in the absence of signs of fetal compromise and contractions, a cervical cerclage can be maintained for 48 hours.	E
Question 98 Gestational thrombocytopenia is characterized by: A. a drop in platelet count below 70,000/mm³. B. the presence of thrombocytopenia outside of pregnancy. C. co-occurrence of thrombocytopenia in the newborn. D. the appearance of thrombocytopenia in the second half of pregnancy. E. the presence of signs of a bleeding diathesis.	D
Question 99 For a patient with a previous lower segment cesarean section who expresses a desire to attempt a vaginal birth, the following interventions are possible: 1) pre-induction of labor with oxytocin; 2) continuous cardiotocographic monitoring during labor; 3) pre-induction of labor with a Foley catheter; 4) augmentation of uterine contractions with oxytocin; 5) a previous cesarean section is always an indication for another one. The correct answer is: A. 2, 3, 4. B. 1, 2, 4. C. only 5. D. 2, 4. E. 1, 4.	A
Question 100 When using prothrombin complex concentrate (PCC) for obstetric hemorrhage, one must consider: A. blood group compatibility. B. increased risk of intravascular coagulation. C. high risk of transfusion-related acute lung injury (TRALI). D. increased risk of transmitting infectious agents. E. all of the above.	B
Question 101 Indicate the true statement regarding twin reversed arterial perfusion (TRAP) sequence: A. in the umbilical artery of the acardiac fetus, blood flows from the placenta to the fetus. B. the blood in the umbilical vein of the acardiac fetus is better oxygenated than the blood in the umbilical vein of the pump twin. C. the blood in the umbilical vein of the acardiac fetus is better oxygenated than the blood in the umbilical artery of that fetus. D. the blood in the umbilical artery of the pump twin is better oxygenated than in the umbilical vein of that twin. E. in the umbilical artery of the pump twin, blood flows from the placenta to the fetus.	C
Question 102 A loop electrosurgical excision procedure (LEEP) of the cervix in a pregnant woman can be performed: A. in case of suspected progression of observed HSIL CIN lesions. B. in case of suspected invasive cancer, when there are diagnostic doubts based on previous examinations. C. up to the 15th week of pregnancy in a hospital setting. D. answers A, B, and C are true. E. the procedure is absolutely contraindicated during pregnancy.	B
Question 103 Indicate the true statement regarding the enlargement of the ventricular system of the fetal central nervous system (ventriculomegaly): A. it is diagnosed when the measurement of the posterior horn of the lateral ventricle is above 5 mm. B. fetal ventriculomegaly is also called "hydrocephalus". C. the treatment of choice for ventriculomegaly is the placement of a shunt into the fetal lateral ventricle, which improves the prognosis for the newborn. D. a finding of ventriculomegaly in a fetus requires a detailed anatomical assessment of the entire fetal CNS and consideration of testing for genetic defects and TORCH infections. E. all of the above.	D
Question 104 Indicate the true statements regarding parvovirus B19 infection: 1) virus replication occurs in erythropoietic progenitor cells; 2) the greatest risk of fetal complications occurs as a result of infection in the second trimester; 3) the greatest risk of fetal complications occurs as a result of infection in the first and third trimesters; 4) the most common fetal sign in case of infection is hydrops fetalis; 5) hydrops fetalis resulting from the infection is always associated with fetal anemia. The correct answer is: A. 1, 2, 4. B. 1, 2, 5. C. 1, 3, 4. D. 1, 3, 5. E. 2, 4, 5.	A
Question 105 Indicate the true statements regarding hydatidiform mole: 1) in a complete mole, there is no fetal development; 2) in a pregnant woman with a mole, hyperthyroidism or hyperemesis gravidarum often occurs; 3) a complete hydatidiform mole has a diploid number of chromosomes; 4) a partial mole is associated with a high risk of developing persistent trophoblastic disease; 5) in the therapy of a complete mole, oxytocin or prostaglandins should not be used to evacuate the uterine cavity. The correct answer is: A. 1, 2, 3. B. 1, 2, 3, 4. C. 1, 3, 4, 5. D. 1, 2, 3, 5. E. all of the above.	D
Question 106 In which situation can the double test (PAPP-A and free beta-hCG) between 10-14 weeks of gestation for first-trimester genetic risk analysis be omitted? 1) in a monochorionic, monoamniotic twin pregnancy; 2) in the absence of a fetal heartbeat; 3) if NIPT was previously performed; 4) if a fetal structural defect is found. The correct answer is: A. 1, 2, 3. B. only 1. C. 1, 4. D. 1, 3. E. 2, 3, 4.	E
Question 107 Prematurity does not affect the following parameters of the fetal biophysical profile: 1) fetal tone; 2) fetal movements; 3) fetal breathing movements; 4) amniotic fluid volume (pocket size); 5) CTG trace. The correct answer is: A. 1, 3. B. 2, 4. C. 1, 4. D. 2, 3. E. 3, 5.	C
Question 108 What is the management of choice for stage 3 Twin-to-Twin Transfusion Syndrome (TTTS) in a monochorionic twin pregnancy at 28 weeks of gestation, without signs of cardiac failure in the recipient? 1) administration of digitalis glycosides; 2) fetoscopy; 3) amnioreduction; 4) delivery after preparation (administration of steroids and neuroprotection). The correct answer is: A. 1, 2. B. 2, 3. C. 3, 4. D. 2, 4. E. 1, 3, 4.	D
Question 109 In the treatment of coagulopathy secondary to massive postpartum hemorrhage during a cesarean section for placenta previa, after placing a balloon tamponade, the following preparations are used: 1) blood products: packed red blood cells, fresh frozen plasma, cryoprecipitate in a 1:1:1 ratio; 2) tranexamic acid at a dose of 1-2 g intravenously; 3) PCC - (Beriplex, Octaplex, Prothromplex) - prothrombin complex concentrate; 4) RIASTAP - fibrinogen concentrate at a dose of 2-3 g intravenously; 5) rFVIIa - recombinant activated factor VII at a dose of 40 - 120 µg/kg body weight; 6) nitroglycerin at a dose of 50 µg intravenously; 7) magnesium sulfate at a dose of 4 - 6 g intravenously. The correct answer is: A. all of the above. B. 1, 2, 5, 6, 7. C. 1, 2, 4, 5, 7. D. 1, 2, 3, 4, 5. E. 1, 2, 4, 6, 7.	D
Question 110 According to current recommendations, in the first trimester of pregnancy, to exclude iron deficiency and iron deficiency anemia, it is recommended to perform: A. a complete blood count assessing hemoglobin level and mean corpuscular hemoglobin. B. a complete blood count assessing hemoglobin level and mean corpuscular volume, and transferrin concentration. C. a complete blood count assessing hemoglobin level and mean corpuscular volume, and iron concentration. D. a complete blood count assessing hemoglobin level and mean corpuscular volume, and ferritin concentration. E. a complete blood count assessing hemoglobin level and mean corpuscular volume, and folic acid concentration.	D
Question 111 Elements subject to detailed ultrasound assessment in the case of myelomeningocele (MMC) are: A. the upper and lower level of the spinal lesion/defect. B. the presence of a hernial sac. C. spinal deformities (scoliosis, kyphosis). D. colpocephaly, banana sign, lemon sign. E. all of the above.	E
Question 112 Due to the increased risk of venous thromboembolism, thromboprophylaxis is an important element of post-cesarean section management. Low-molecular-weight heparin should be used only for a short term (2-7 days) in the postpartum period for the patients listed below, EXCEPT for: A. a 40-year-old, after her fourth cesarean section. B. a 37-year-old with a BMI >30 kg/m². C. a patient who smokes cigarettes, with coexisting varicose veins of the lower limbs. D. a patient who used LMWH at a prophylactic dose throughout the entire pregnancy. E. a patient with asymptomatic thrombophilia.	D
Question 113 Induction of labor in a G2 P1 patient at 38+5 weeks with gestational hypertension should be refrained from in all of the following clinical situations, EXCEPT: A. active cervical cancer. B. status post shoulder dystocia in a previous delivery (current EFW 3400 g). C. status post laparoscopic myomectomy of an intramural fibroid from the uterine body. D. status post injury to the internal and external anal sphincter in a previous delivery. E. active HSV-2 infection.	B
Question 114 Prelabour rupture of membranes means rupture of membranes without the onset of regular uterine contractions. It can occur at term or before 37 weeks of gestation. PPROM carries an increased risk of intrauterine infection. The clinical signs of intrauterine infection include the presence of two of the following, EXCEPT: A. leukocytosis > 15 G/L. B. fetal tachycardia >160 bpm. C. uterine tenderness. D. maternal tachycardia < 100 bpm. E. body temperature >38 °C.	D
Question 115 Indicate the false statement regarding postpartum uterine tamponade: A. it is one of the treatment methods for uterine atony. B. it should be used after an attempt at pharmacological treatment. C. it can be maintained for 24 - 48 hours. D. it can be performed with balloons - a Foley catheter, Bakri balloon, Sengstaken-Blakemore tube. E. it can be done with sterile gauze packs or swabs secured with a surgical drape.	C
Question 116 The adverse effects of prostaglandins used for induction of labor or abortion include: 1) dyspnea; 2) headache and dizziness; 3) tachycardia; 4) increase in body temperature; 5) increase in blood pressure; 6) diarrhea. The correct answer is: A. 1, 2, 3, 4. B. 3, 4, 5, 6. C. 1, 2, 4, 6. D. 2, 3, 5, 6. E. all of the above.	E
Question 117 For a patient at 25 weeks of a singleton pregnancy with a maximum vertical pocket (MVP) of 11 cm, the following additional tests should be ordered: A. an ultrasound examination at a referral center to exclude fetal developmental abnormalities and markers of chromosomal aberrations. B. an oral glucose tolerance test (OGTT) to exclude diabetes. C. after excluding other possible causes of polyhydramnios - amniocentesis with fetal karyotyping and testing for cytomegalovirus and toxoplasma DNA. D. answers A, B, and C are true. E. answers A and B are true.	D
Question 118 Placenta accreta spectrum (PAS) disorders are pathological conditions of pregnancy, resulting from abnormal trophoblast invasion, characterized by difficulties in placental separation after childbirth. Risk factors for PAS disorders include: 1) pregnancy resulting from assisted reproductive technology; 2) multiple pregnancy; 3) history of manual removal of the placenta; 4) history of hysteroscopy; 5) history of endometritis; 6) postpartum hemorrhage in a previous pregnancy; 7) bicornuate uterus; 8) use of an intrauterine device before pregnancy. The correct answer is: A. 3, 5, 7. B. 3, 4, 5, 7. C. 1, 2, 6, 8. D. 1, 3, 5, 7, 8. E. all of the above.	E
Question 119 Indicate the false statement regarding sulprostone: A. it is a PGF2-alpha analogue. B. it is a second-line drug in the treatment of postpartum hemorrhage. C. the recommended dose is 500 µg dissolved in 500 ml of solution for intravenous infusion. D. a common adverse effect is a local reaction at the infusion site. E. the maximum daily dose is 1500 µg.	A
Question 120 Based on the recommendations of the National Medicines Institute, for patients with PPROM who are allergic to penicillin, the recommended antibiotic therapy regimen is: A. azithromycin 1 g p.o. once + ampicillin 2 g i.v. every 6 hours for 48 hours, then amoxicillin 500 mg every 8 hours p.o. for 5 days. B. azithromycin 1 g p.o. once + clindamycin 900 mg every 8 hours i.v. for 48 hours, then clindamycin 300 mg every 6 hours p.o. for 5 days. C. azithromycin 1 g p.o. once + clindamycin 900 mg every 8 hours i.v. for 48 hours, then clindamycin 300 mg every 8 hours p.o. for 5 days. D. azithromycin 1 g p.o. once + clindamycin 900 mg every 8 hours i.v. for 48 hours, then clindamycin 900 mg every 6 hours p.o. for 5 days. E. azithromycin 1 g p.o. once + clindamycin 900 mg every 8 hours i.v. for 48 hours, then clindamycin 900 mg every 8 hours p.o. for 5 days.	C
